# Nanoarchitectonics of Nanoporous Carbon Materials in Supercapacitors Applications

**DOI:** 10.3390/nano10040639

**Published:** 2020-03-29

**Authors:** Rekha Goswami Shrestha, Subrata Maji, Lok Kumar Shrestha, Katsuhiko Ariga

**Affiliations:** 1 International Center for Materials Nanoarchitectonics (WPI−MANA), National Institute for Materials Science (NIMS), 1−1 Namiki, Tsukuba 305−0044, Japan; MAJI.Subrata@nims.go.jp (S.M.); SHRESTHA.LokKumar@nims.go.jp (L.K.S.); 2 Graduate School of Frontier Sciences, The University of Tokyo, 5-1-5 Kashiwanoha, Kashiwa, Chiba 277−8561, Japan

**Keywords:** carbon, energy storage, fullerene, nanoarchitectonics, nanoporous

## Abstract

High surface area and large pore volume carbon materials having hierarchical nanoporous structure are required in high performance supercapacitors. Such nanoporous carbon materials can be fabricated from organic precursors with high carbon content, such as synthetic biomass or agricultural wastes containing cellulose, hemicellulose, and lignin. Using recently developed unique concept of materials nanoarchitectonics, high performance porous carbons with controllable surface area, pore size distribution, and hierarchy in nanoporous structure can be fabricated. In this review, we will overview the recent trends and advancements on the synthetic methods for the production of hierarchical porous carbons with one- to three-dimensional network structure with superior performance in supercapacitors applications. We highlight the promising scope of accessing nanoporous graphitic carbon materials from: (i) direct conversion of single crystalline self-assembled fullerene nanomaterials and metal organic frameworks, (ii) hard- and soft-templating routes, and (iii) the direct carbonization and/or activation of biomass or agricultural wastes as non-templating routes. We discuss the appealing points of the different synthetic carbon sources and natural precursor raw−materials derived nanoporous carbon materials in supercapacitors applications.

## 1. Introduction

**Scheme 1:**
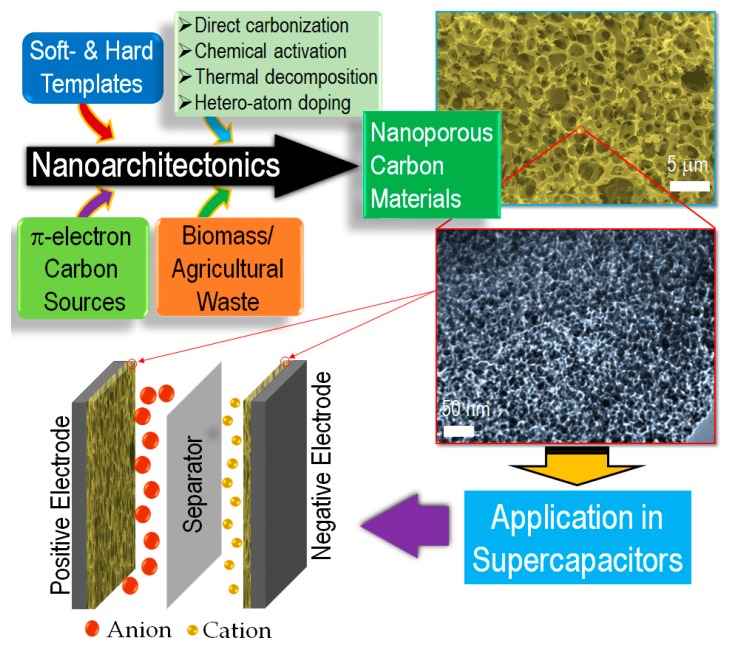
Schematic illustration of the nanoarchitectonics concept in which nanoporous carbon materials are fabricated by different synthetic approaches from various synthetic and natural biomass as carbon sources for supercapacitors applications.

Various social demands including environmental problems [1,2,3,4,5], energy managements [6,7,8,9], device development [10,11,12,13,14], and biomedical applications [15,16,17,18,19] have been handled with various scientific and technological developments. This includes organic synthesis [20,21,22,23,24], polymer synthesis [25,26,27], energy production [28,29,30,31], energy storage [32,33,34], sensing [35,36,37,38,39], photocatalysis [40,41,42,43], organic synthetic catalyst [44,45,46,47], self−assembly [48,49,50,51], and biological investigation [52,53,54] in addition to analytical approach [55,56,57] and theoretical consideration [58,59,60,61]. In order to develop highly efficient systems, synthesis and organization of nanomaterials becomes much more important [62,63,64,65]. Especially, an emerging concept, nanoarchitectonics, has crucial roles for these challenges to fabricate functional materials from nano-units [66,67]. This concept was initially developed by Masakazu Aono [68,69] to combine nanotechnology with the other research fields such as supramolecular chemistry and nanofabrications [70,71]. This concept has been applied to many research fields such as materials synthesis [72,73,74], structure organization [75,76], catalysts [77], sensing [78,79], device [80,81], energy [82,83], environment [84], and biological applications [85,86]. In this review, recent developments on important subject, nanoarchitectonics of nanoporous carbon materials for supercapacitors applications, are overviewed. Different synthetic methods using different starting materials and general design and basic principles for the production of nanoporous carbon materials for supercapacitors applications through nanoarchitectonics concept (Scheme 1) are illustrated.

Supercapacitors (SCs) are the current state-of-the-art electrochemical energy storage systems that have been extensively used in hybrid electric vehicles, portable electronic devices, memory backup systems, and also in industrial energy and power managements. Compared to lithium-ion batteries, SCs are more convenient devices for the energy storage because of their high power density (410 kW kg^−1^), extraordinary cycling stability (>10 000 cycles), low-cost, low internal resistance, fast charging−discharging dynamics, and high rate capability [87]. Based on the energy storage mechanism, there are two distinct types of supercapacitors called electrical double-layer capacitors (EDLCs) and pseudocapacitors (PCs). In EDLCs, the energy storage mechanism involves the formation of electrical double layer charges at the electrode/electrolyte interface due to electrostatic adsorption of electrolyte ions. As a result, the EDLCs allow fast charging–discharging rate. On the other hand, in PCs reversible Faradic reactions occur at the electrode surface followed by the charge transfer and, thus, they offer higher specific capacitance and energy density compared to those of EDLCs [88]. Many pseudocapacitive materials including transition metals, their oxides, rate-earth metal oxides, and conducting polymers have been explored as electrode materials of the PCs [89]. For example, manganese oxides (MnO_2_ and Mn_2_O_3_), cobalt oxides (CoO and Co_3_O_4_), nickel oxides (NiO and Ni_2_O_3_), cerium oxides (CeO_2_), vanadium pentoxide (V_2_O_5_), and iron oxide (Fe_2_O_3_) are commonly used electrode materials for PCs [90]. Recently, hybrid supercapacitors that essentially comprise of EDLC and PC materials are in common practice for better energy storage. Hybrid supercapacitive materials generally include binary or ternary composites of metal oxides and also carbon composites with metal oxides [91]. 

Despite the aforementioned several advantages, SCs suffer from the delivery of low energy density (1−10 W h kg^−1^) compared to the lithium-ion batteries (160 W h kg^−1^) [92]. Therefore, it is yet a key challenge to fabricate supercapacitive materials that can deliver high energy density sustaining other intrinsic properties of high power density, long cycling stability and fast charging−discharging dynamics. Special and extensive efforts have been given to solve this issue by the fabrication of novel supercapacitor electrode materials, which can achieve high specific capacitances ‘*C_s_*’, and also can be operational over a wide potential window ‘*V*’. This is because the energy storage is proportional to *C_s_V*^2^ [93]. Therefore, the design of novel functional nanoporous carbon materials with high specific capacitances is expected to facilitate the assembly of effective energy storage systems that can deliver high energy densities. Another strategy to achieve high energy density is the assembly of asymmetric supercapacitors. In this case, two different electrode materials with different potential windows can gain a broader operating voltage [94].

Nanoporous carbon materials are one of the foremost electrode materials used in commercial SCs because of low-cost, outstanding cycling stability, and wide operating voltage window. Fullerenes, carbon nanotubes, graphene, and carbon nanohorns are the well-known nanocarbon materials that have been explored in energy storage, energy conversion, sensing, separation, purification, and also in biomedical technology [95,96,97]. These materials show unique physicochemical and optoelectronic properties and, thus, are used in the functional system design. In supercapacitor applications, hierarchical nanoporous carbons materials that exhibit high surface area and well established porosity with large pore volumes are highly demanded [98,99,100]. Therefore, attention has been paid to fabricate novel carbon materials with smart properties and functions. Nanoporous carbon materials are mainly carbonaceous materials and can be fabricated by the pyrolysis of different synthetic carbon sources and metal organic frameworks, templating self-assembled soft and hard nanomaterials, and natural bio-masses or agricultural wastes containing cellulose, hemicellulose, and lignin. It has been found that depending on the synthetic conditions, preparation method, and type of precursors or carbon sources used, porous carbon materials with different pore sizes (micro−macro via mesopores) can be fabricated. Contrary to the conventional agricultural wastes including coconut shell, corncob, bamboo, pitch stone, etc. researchers have been exploring novel precursors such as biowaste chicken eggshell containing high amount of calcium carbonate for the fabrication of new supercapacitive materials [101,102]. For designing such high performance materials with tunable surface textural properties, nanopores engineering, control of hierarchical nanoporous structure, and novel supercapacitive materials, concept of nanoarchitectonics would be very useful.

In this review, we will overview recent trends and the advancements of the synthetic methods for the fabrications of hierarchical porous carbons materials with one- to three-dimensional network structure for supercapacitors applications. We will also highlight the promising scope of accessing nanoporous graphitic carbon materials obtained from the direct conversion of single crystalline self-assembled fullerene nanomaterials and metal organic frameworks, hard- and soft-templating routes and also the direct carbonization and/or activation of biomass or agricultural wastes as non-templating routes. 

## 2. Nanoporous Carbons Materials

Hierarchical porous carbon materials having 3D network structure have been used in a wider range of applications ranging from supercapacitors, lithium-ion batteries, fuel cells, solar cells, hydrogen storage, gas sensing, separation of toxic gases or ionic species, etc. High surface areas, large pore volumes, good electrical conductivity, and excellent chemical stabilities are the unique physicochemical properties, which made nanoporous carbons of high potential impact in the targeted applications such as supercapacitors. However, it is yet challenging to fabricate new porous carbon materials with controlled-dimension and porosity, tunable surface area, hierarchical, and interconnected pore network structures. In the following section, we will overview the recent trends and progressions in the fabrication of nanoporous carbon materials from different synthetic carbon sources such as polymers, self-assembled materials, and biomass or biopolymers and their applications in supercapacitors. 

### 2.1. Nanoporous Carbons from Self-Assembled Fullerene Crystals

Supramolecular assemblies of fullerenes (C_60_ or C_70_) through −stacking interactions allow shape- and size-controlled nano–micro-crystals fabrication in solution, on a solid substrate and at air−liquid or liquid−liquid interfaces. Self-assembled fullerenes into well-ordered 1D, 2D, or 3D forms exhibit enhanced optoelectronics properties [103]. Therefore, these nanostructured fullerene-based materials have been used to design novel functional systems useful in optoelectronic and spintronic devices, organic semiconductors, and biomedicines. Several synthetic methods have been reported to synthesize fullerene nanocrystals and morphology of the prepared crystals was found to be method dependent. However, of several methods, the method developed by Miyazawa and co-workers called liquid−liquid interfacial precipitation (LLIP) has become one of the versatile methods to control the morphology of fullerene assemblies from zero to higher dimensions [104]. For 1D fullerene materials, Shrestha and co-workers realized the first example of the ultra-rapid formation of fullerene nanorods [105]. Crystal formation completed within a few seconds due to the instantaneous intermixing of poor solvent to fullerene (methanol) and good solvent (mesitylene). Besides, the method is suitable for the scale-up synthesis of 1D fullerene materials. LLIP method has been further extended to produce mesoporous fullerene crystals, hierarchical fullerene cubes with sensing antenna, and also the fullerene cubes with an open hole in each face of the cube, which can selectively recognize spherical carbon particle having graphitic nature over the resorcinol−formaldehyde polymer resin particle of similar shape and size. 

These simply prepared fullerene nanomaterials could be directly converted into high surface area nanoporous carbon materials upon high-temperature heat-treatment. As demonstrated by Shrestha and co-workers [106] fullerene C_60_ nanorods or nanotubes could be transformed into carbon nanorods or nanotubes by heat-treatment at 2000 °C in vacuum. During the heat-treatment, thermally unstable pentagon rings of the fullerene molecules are disrupted followed by breaking of the hexagonal rings and eventually the formation of highly graphitic structures with high thermal stability was observed. The Brunauer−Emmett−Teller (BET) surface area of carbon nanorods and nanotubes were ca. 1600 m^2^ g^−1^, 1650 m^2^ g^−1^, respectively, which demonstrate that the fullerene crystals derived carbon materials (both nanoporous carbon rods and tubes) display high surface area comparable to that of the commercial activated carbons (1000−1500 m^2^ g^−1^), mesoporous carbons prepared by templating method (500−1000 m^2^ g^−1^), and commercial single-walled carbon nanotubes (1300 m^2^ g^−1^). Interestingly, they have observed that thermally converted carbon nanorods or nanotube exhibit stacking of graphene layers (maximum of six layers) with interlayer spacing ca. 0.35 nm. Due to the enhanced electrochemically accessible surface area, these carbon materials showed excellent electrochemical supercapacitors performance giving specific capacitances of 145.5 F g^−1^ (carbon tubes) and 132.3 F g^−1^ (carbon rods) at 5 mV s^−1^. 

**Figure 1:**
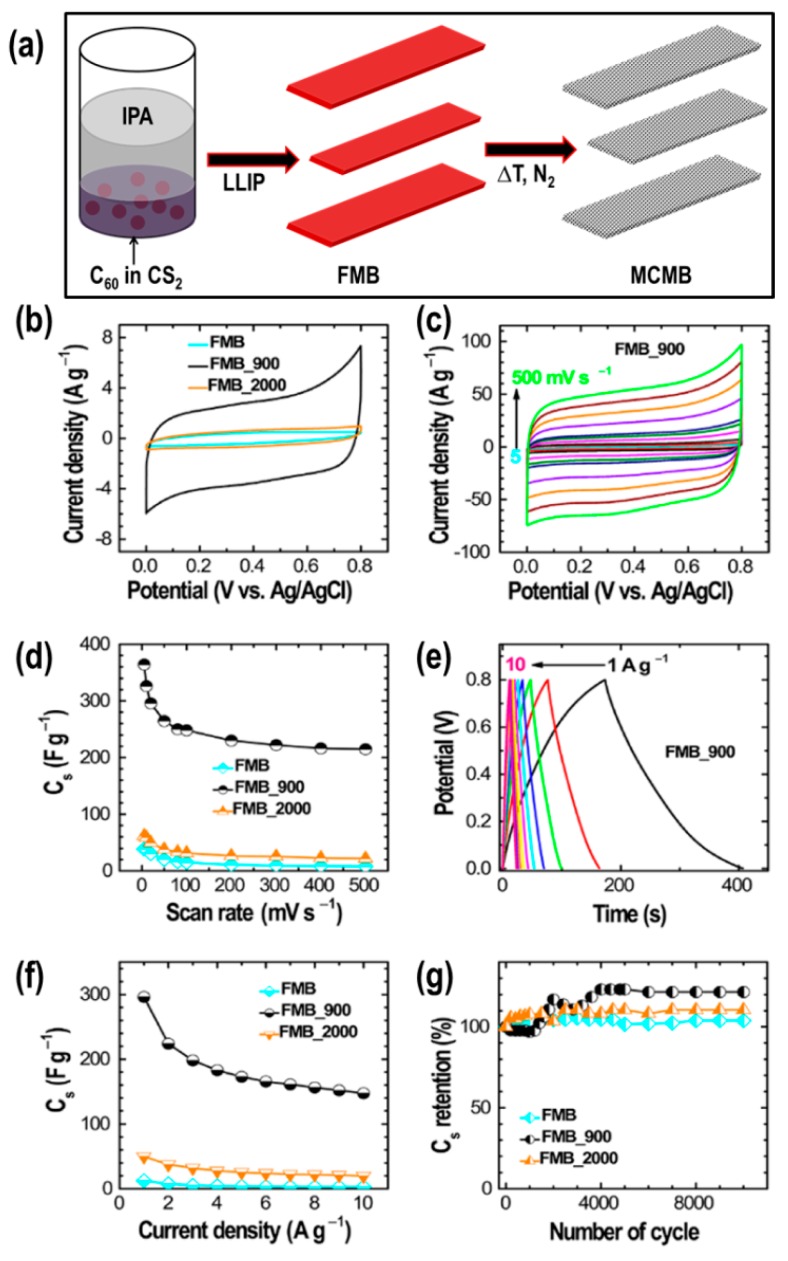
(**a**) Illustration of fullerene C_60_ microbelts (FMBs) crystals synthesis and their conversion into mesoporous carbon microbelts (MCBs), (**b**) Cyclic voltammograms (CV) of the prepared materials at a fixed scan rate of (5 mV s^−1^), (**c**) CV curves vs. scan rate as a typical example of the optimal sample (FMB_900). (**d**) Comparison of calculated specific capacitance of prepared samples (FMB, FMB_900, and FMB_2000). (**e**) Typical example of charge−discharge (CD) curves vs. current density (1−10 A g^−1^). (**f**) Variation of calculated specific capacitance vs. current densities showing the high rate performance of mesoporous carbon microbelts, and (**g**) Capacitance retention up to 10,000 charge−discharge cycles. Reproduced from Reference [109]. Copyright American Chemical Society, 2017.

Similarly, Bairi, Shrestha, and co-workers successfully converted mesoporous crystalline fullerene C_70_ microtubes into mesoporous carbon microtubes with graphitic pore walls by heating the fullerene C_70_ microtubes at 2000 °C in vacuum [107]. Here again, the initial 1D hollow structure is sustained even after the heat-treatment and the converted carbon has a mesoporous structure with conjugated *sp*^2^ carbon-rich graphitic frameworks. Due to the enhanced surface properties including surface area and porosity, fullerene-derived mesoporous carbon microtubes showed excellent electrochemical performance in aqueous electrolyte (1 M H_2_SO_4_) in a three-electrode system specific capacitance ca. 212.2 F g^−1^ at 5 mV s^−1^ and 184.6 F g^−1^ at a current density of 0.5 A g^−1^ followed by good rate performance at high current density [107]. Shrestha and co-workers have also extended the method and fabricated surfactant/fullerene hybrid materials with a unique shape of sugar-candy type crystals, which upon heat-treatment could be converted into graphitic carbons that exhibit enhanced surface area [108]. As a result, the material achieved a specific capacitance of 175 F g^−1^ at 5 mV s^−1^ and 115 F g^−1^ at 1 A g^−1^. The obtained values are higher (20 times) than pristine C_60_ or sugar-candy type crystals before the heat-treatment. Furthermore, the rate performance of the electrode was good as it could sustain about 45% capacitance at a high current density of 10 A g^−1^ and also show long cycle stability, 95 % capacitance was retained after 1000 charge−discharge cycles [108]. Tang, Ji, and co-workers have demonstrated large size pseudo 2D fullerene C_60_ microbelts having lengths and widths ca. 172 and 26 µm, respectively [109]. Such unusually large-sized microbelts were produced using LLIP method from isopropyl alcohol (IPA) fullerene solution in carbon disulfide (CS_2_) at ambient temperature (Figure 1). They have also demonstrated that the fullerene C_60_ microbelt morphology could sustain 900 °C temperature with the resultant product being converted into quasi 2D mesoporous carbon microbelts (MCMBs) that exhibit a high surface area and robust mesoporous framework structure. As a result, the material achieved very high specific capacitances ca. 360 F g^−1^ at 5 mV s^−1^ and 290 F g^−1^ at 1 A g^−1^. The electrode showed good rate performance retaining 49% capacitance at a high scan rate of 10 A g^−1^ followed by super cyclic stability without any capacity loss after 10 000 charge−discharge cycles [109]. 

**Figure 2:**
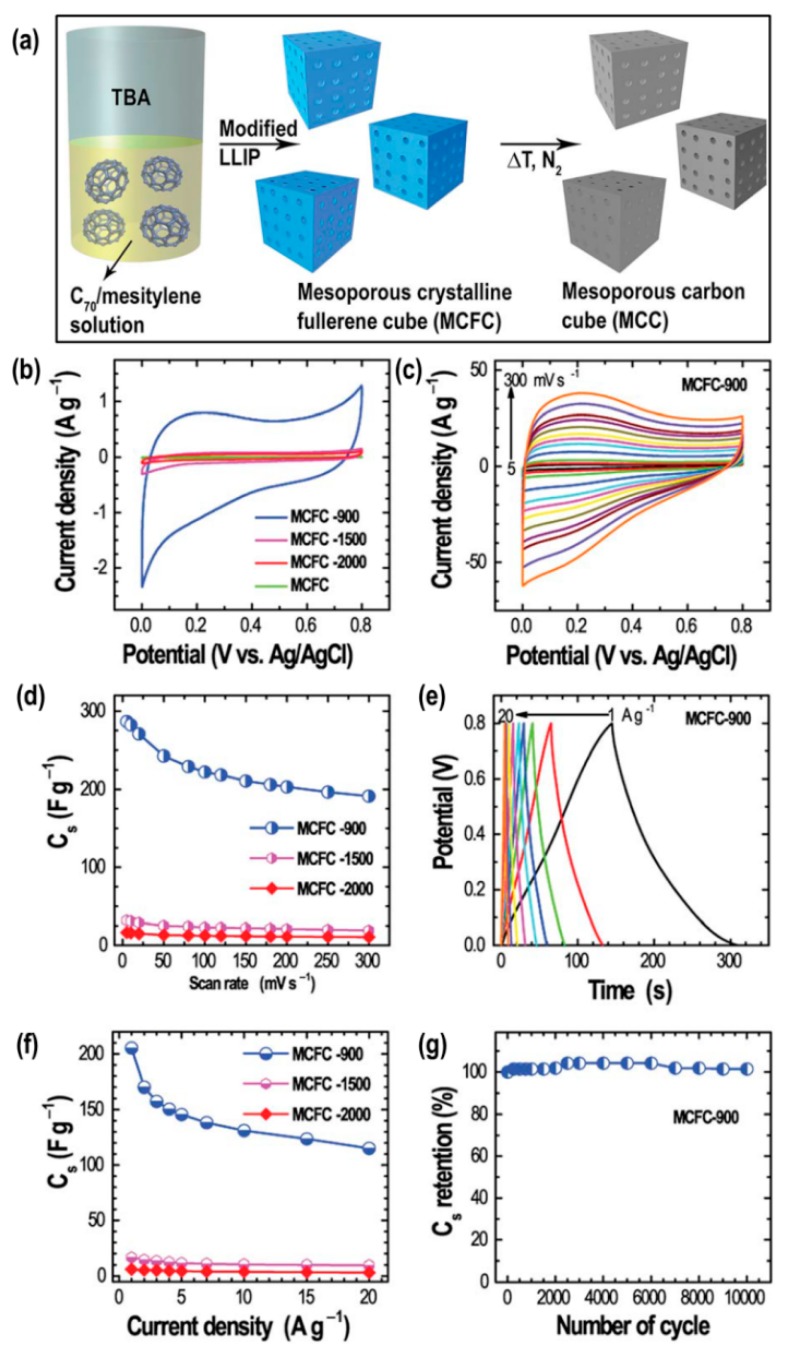
(**a**) Illustration of the synthesis of mesoporous crystalline fullerene cube (MCFC) and mesoporous carbon cubes. (**b**) Comparison of CV curves of carbon samples at 5 mV s^−1^, (**c**) CV curves vs. scan rate for optimal sample MCFC−900, (**d**) Calculated specific capacitance vs. scan rates for the prepared samples, (**e**) CD curves vs. current density for optimal sample MCFC−900 showing electrical double-layer capacitor (EDLC) behavior, (**f**) Calculated specific capacitance vs. current densities, and (**g**) Cycle stability performance of MCFC−900 recorded at 7 A g^−1^. Reproduced by permission of The Royal Society of Chemistry (RSC) from Reference [110].

Very recently, Bairi, Shrestha, and co-workers have extended the thermolysis method to 3D mesoporous crystalline fullerene C_70_ cubes prepared by LLIP method and obtained mesoporous carbon cubes (MCCs) that displayed high specific surface area (Figure 2) [110]. High-resolution transmission microscopy imaging revealed well-developed mesoporous carbon structure. The newly prepared 3D mesoporous carbon cubes performed well in a three-electrode electrochemical system. Although the calculated specific capacitances (286 F g^−1^ (at 5 mV s^−1^) and 205 F g^−1^ (at 1 A g^−1^)) was lower than that of the mesoporous carbon microbelts, capacitance retention of the carbon cubes was found far better sustaining about 66% capacitance at a high scan rate of 300 mV s^−1^ indicating a fast electrolyte ion diffusion at the electrode surface. Furthermore, they have also observed high rate performance and long cycle stability of the electrode with the retention of 56.0% capacitance at 20 A g^−1^ and without any loss capacitance after 10 000 charge−discharge cycles [110]. 

All these electrochemical results demonstrate that the hierarchical nanoporous carbons derived from self-assembled fullerene crystals could be encouraging supercapacitor electrode materials for high energy storage and high-performance supercapacitors devices (high specific capacitance, good rate capability, and long cycle life). As mentioned earlier, the production of micro–meso-porous carbons having graphitic pore walls is a challenging task, the thermally converted nanoporous carbons whose porosity could be tuned from micro–macro-pores via mesopores having robust graphitic carbon walls using fullerene crystals as the starting material is expected to be advantageous for the design of high-performance advanced supercapacitor devices. 

### 2.2. Nanoporous Carbons from Metal−Organic Frameworks

Metal−organic frameworks (MOFs) have been regarded as the new class of porous materials that exhibit extremely high surface areas and large pore volumes compared to other porous materials such as activated carbons. Extensive efforts have been given to utilize the MOFs for different applications including gas adsorption, heterogeneous catalysis, and energy storage. However, MOFs have restricted applications in supercapacitors as they have low electrical conductivity and low stability. MOFs contain a large amount of carbon, therefore, they can serve as excellent precursors for the synthesis of carbon materials having high surface area and high porosity. Moreover, porous MOFs have permanent nano-size cavities and open channels accessible to small molecules. Therefore, MOFs function as an efficient template to fabricate nanoporous carbon materials. Kim, Yamauchi, and co-workers have highlighted the recent development of the fabrication of 3D MOF-derived porous carbon materials in energy storage applications including supercapacitor and also discussed the significance of the properties of the parent MOF and the derived carbons [111]. 

The preparation of MOFs is possible with a large number of mixtures of organic and inorganic elements and is based on basic fundamentals of coordination chemistry. Free control of their pore architectures, pore size distributions, pore volumes, and surface areas is possible. The unique surface textural properties of MOF are sustained in the MOF-derived nanoporous carbons, which make the prepared carbon materials very attractive in technological applications including sensing, separation, purification, energy storage, and conversion. Furthermore, MOF-derived carbons are advantageous due to ease of fabrication method and rather precise control of the porosity and surface areas. 

Liu, Xu, and co-workers have demonstrated the first example that MOF functions as a template to prepare nanoporous carbon materials with the high specific surface that shows superior EDLC property [112]. They have used MOF-5 framework (Zn_4_O(OOCC_6_H_4_COO)_3_) having a three-dimensional intersecting channel system with cavity diameter 18 Å as a template and furfuryl alcohol (FA) as carbon precursor, respectively. First, they have polymerized furfuryl alcohol/MOF-5 composite by heating the degassed MOF-5 at 150 °C for 48 h under the atmosphere of FA vapor followed by the carbonization of the composite at 1000 °C for 8 h with a constant flow of Ar gas. The obtained carbon materials exhibit excellent surface textural properties. The BET surface area and pore volume are ca. 2872 m^2^ g^−1^ and 2.06 cc g^−1^, respectively. They have also found that surface area decreased drastically in a sample obtained by carbonizing at 800 °C. The BET surface area and pore volume were ca. 417 m^2^ g^−1^ and 0.63 cc g^−1^, respectively demonstrating the fact that the carbonization temperature is critical for the structural evolution of the resulting carbons. The MOF-5 derived carbon performed excellently in the electrochemical energy storage system and the electrode material achieved a high specific capacitance of 312 F g^−1^ at 1 mV s^−1^. Furthermore, the capacitance of the electrode is maintained as high as 258 F g^−1^ at a high current density of 250 mA g^−1^ in galvanostatic charge−discharge measurements showing possibilities for the fabrication of several newer nanoporous carbon materials for high energy storage applications [112]. 

**Figure 3:**
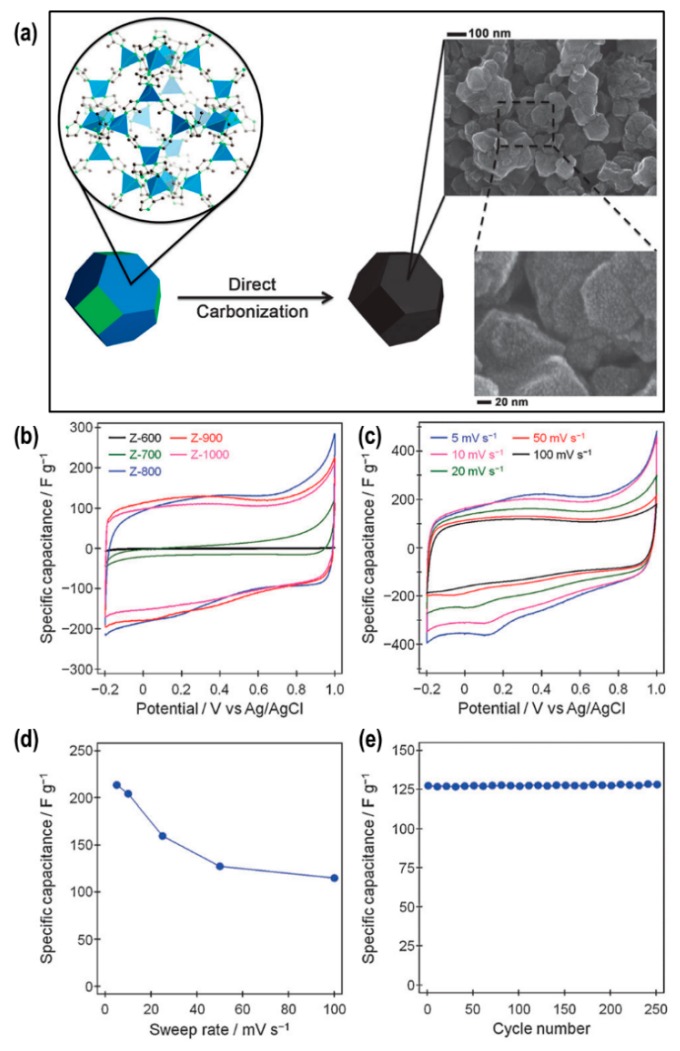
(**a**) Schematic demonstration of ZIF-8−derived nanoporous carbons with SEM images of different magnifications. (**b**) CV curves a scan rate of 50 mV s^−1^ for different nanoporous carbon samples, (**c**) Corresponding CV curve vs. scan rate for the optimal sample Z−900, (**d**) Decay of specific capacitance vs. scan rate for Z−900, and (**e**) Corresponding cycle life at 50 mV s^−1^. Reproduced by permission of The Royal Society of Chemistry (RSC) from Reference [113], 2012.

Yamauchi and co-workers have also developed rather a simple method of direct carbonization of commercial zeolitic imidazolate framework (ZIF-8) to prepare carbon materials with nanopore architecture having amorphous carbon frameworks [113]. In their method, they did not employ any carbon sources (Figure 3). BET surface areas of the ZIF-8-derived nanoporous carbon materials could be tuned from 520 to 1110 m^2^ g^−1^ depending on the carbonization temperature. The ZIF-8 derived nanoporous carbon materials achieved high volumetric capacitance of 200 F g^−1^ at 5 mV s^−1^ in 0.5 M H2SO4 aqueous electrolyte without losing capacitance after 250 cycles at 50 mV s^−1^ demonstrating good cycle stability of the electrode [113].

Similarly, another MOF ZIF-67 which comprise of cobalt ions coordinated with four imidazole rings has been converted into good electrical conductivity graphitized carbon frameworks because the cobalt present in ZIF-67 catalyzes the graphitization during carbonization [114]. This shows that for high surface area carbon material preparation, ZIF-8 is a better choice. However, for conductive carbon material production ZIF-67 could be the better source. Yamauchi and co-workers also proposed a new method for the fabrication of nanoporous hybrid carbon with selective functionalization using ZIF-8@ZIF-67 core−shell structured crystals as the starting material. Firstly, ZIF-8@ZIF-67 crystals were synthesized through a method called seed-mediated growth. Then the obtained material was carbonized at 800 °C to obtain hybrid nanoporous carbon materials comprised of nitrogen-doped carbon and graphitic carbon as core and shell, respectively. Due to the hierarchic superstructure, this new carbon material showed synergistic properties of nitrogen-doping and graphitic carbon structure exhibiting a high specific capacitance of 270 F g^−1^ at 2 A g^−1^. Their study showed the possibility of bridging diverse carbon-based materials with a large number of MOFs and nanoarchitectonics of carbon materials with targeted functionalities and applications [114]. Very recently, Kim and co-workers reported the room-temperature synthesis of hybrid MOFs with different Zn^2+^/Co^2+^ ratios using deionized water as a reaction medium to increase the product yield [115]. Hybrid MOF was directly carbonized to obtain nanoporous carbons, however, the surface area of the prepared carbon decreased. The surface area could be increased from 655 to 1417 m^2^ g^−1^ by KOH activation. Furthermore, during activation cobalt nanoparticles were converted into Cobalt oxides (CoO and Co_3_O_4_). These activated carbons display enhanced specific capacitance due to both EDLC (contribution from carbon) and pseudocapacitor (contribution from cobalt oxides) behavior. This method could be utilized to improve the pseudocapacitance of the hybrid materials preserving the surface textural properties (high surface area) of carbons. 

Similarly, Yamauchi and co-workers directly carbonized Al-based porous coordination polymers (Al−PCP) and produced a new type of nanoporous graphitic carbon materials that exhibit ultra-high surface area (BET surface area ca. 5500 m^2^ g^−1^) and large pore volume (4.4 cc g^−1^), which is far better than the commercial activated carbons or mesoporous carbons prepared from templating method [116]. Hill and co-workers have also reported the electrochemical supercapacitive performance of hybrid carbon materials obtained from the direct carbonization of catechol porphyrin-based porous coordination nanorods [117]. They have synthesized catechol-substituted porphyrin[meso−tetrakis(3,4−dihydroxyphenyl)porphyrin] porous coordination polymers constructed from a and found that they exhibit 1D nanorod morphology with moderate surface area (100–400 m^2^ g^−1^) before pyrolysis. However, upon direct carbonization of these polymers at 800 °C in an inert atmosphere formed nanoporous carbon composite materials with metal nanoparticles embedded in the carbon framework, whose surface area was approximately double of the starting materials. As a result, the composite materials exhibit high specific capacitance of 380 F g^−1^ at 1 A g^−1^ demonstrating the potential of porous coordination polymer-derived carbon composite materials as electrode materials in supercapacitor applications. 

### 2.3. Nanoporous Carbons from Hard- and Soft-Templates

The future of supercapacitor devices in industrial-scale application largely depends on the progress and advancement of the fabrication of novel porous carbons with enhanced and optimized porous structures, high specific surface area, excellent electrical conductivity, and also high chemical or electrochemical stability. Several methods have been employed to produce high-quality nanoporous carbon materials from different sources. Of them, templated synthesis is one of the versatile methods for the tailored synthesis of nanoporous carbons with a controllable pore size. Nanopore engineering from micro− to mesopore can be done by turning the size of the template materials. Various inorganic hard solid materials have been templated. Of them, mesoporous silica and zeolites represent the most commonly used templates to produce mesoporous carbon materials with rich surface properties (high surface area and large pore volumes). 

Porous carbon production using the templating method has a long history as it goes back to 1986 when Knox and co-workers used silica gel or porous glass as templates and obtained the porous carbon for the first time [118]. Due to the disordered mesostructure of the template, the produced carbon replica also exhibits the disordered structures. Years later, Ryoo and co-workers reported the first example of ordered carbon molecular sieves [119]. They have used mesoporous silica molecular sieves as template and sucrose as the carbon source and mild carbonization in the presence of sulfuric acid as catalyst resulted in the ordered carbons. Removal of silica template using aqueous sodium hydroxide solution produced the carbon molecular sieves consisting of uniform mesopores (3 nm) arranged in a three-dimensional regular array [119]. Template synthesis essentially consists of pore filling of the template with carbon precursor, carbonization followed by template removal. In order to produce ordered mesoporous carbon, the template should exhibit a three-dimensional porous structure. Using MCM-48, SBA-1, and SBA-15 silica templates, Ryoo and co-workers have successfully synthesized mesoporous carbons with different frameworks (cubic or hexagonal) [120]. Since the pore size distribution of the prepared carbons exhibit high BET surface areas (1800 m^2^ g^−1^) and large pore volumes (1.2 cc g^−1^). 

Many studies have been carried out since then and several interesting ordered mesoporous carbons have been produced. Now the template or nanocasting method has been widely used to tailor the porosity of the replica. Mesoporous carbon molecular sieve, CMK-3 having ordered mesopore structure was first demonstrated by Ryoo and co-workers [121]. They have found that CMK-3 retains the structural symmetry for the silica template, SBA-15. They have also demonstrated that the pore filling degree of the carbon precursor into the hexagonal pore system of SBA-15 determines the structure of the obtained mesoporous carbons [121]. For example, if the pore of the template SBA-15 is completely filled with the precursor, CMK-3 with P6mm symmetry can be formed with the pore structure of CMK-3 the inverse replica of SBA-15 silica. On the other hand, if the pore is partially filled, mesoporous carbons with an array of hollow carbon tubes named CMK-5 are formed. Similarly, large pore size (3–10 nm) mesoporous silica was produced by Ryoo and co-workers, and Zhao and co-workers using triblock copolymer P 123 as a template. The large pore sized silica was then used to produce porous carbon materials having large pores. Zhao and co-workers have also demonstrated rod-like and tube-like mesoporous carbons by the templating method [122,123]. Vinu and co-workers have successfully synthesized novel carbon nanocage, (CKT); mesoporous carbon molecular sieves with large pores having cage type structures using KIT-5 as templates [124,125]. The pore diameters could be controlled by adjusting the ratio of sucrose (carbon source) to KIT-5 (template). They have found that surface area and pore volumes of the carbon nanocage CKT-3(A) are much higher as compared to CMK-3 prepared from SBA-15 [124,125]. 

Another efficient strategy to produce nanoporous carbons is the use of soft materials that can function both as a soft template and starting material. Zhou and co-workers have synthesized high surface area mesoporous carbon nanofibers (BET surface area ca. 1424 m^2^ g^−1^) using self-assembly of triblock copolymers F127 [126]. Due to hexagonally arranged mesoporous channels in the carbon nanofibers, the material offered fast electrolyte ion diffusion due to short diffusion distance and, hence, showed better supercapacitance performance compared to the mesoporous carbon prepared using the same precursor. The specific capacitance of mesoporous carbon fiber in aqueous electrolyte (1 M KOH) was ca. 152 F g^−1^ at 5 mV s^−1^. Several parameters including high surface area, short ion diffusion length, controlled pore size of the mesoporous that is opens to the outer surface have contributed to the excellent performance of 1D mesoporous carbon nanofibers. 

Mokaya and co-workers reviewed the templated synthesis of nanoscale porous carbon where they have nicely summarized different examples of the production of porous carbons by hard and soft templates [127]. They have overviewed accessible wide range of pore sizes of hard or soft templates for the fabrication of ordered nanosize carbons whose surface area and porosity can be optimized. Microwave has also been of great use in the fabrication of mesoporous carbon. He, Qiu, and co-workers synthesized mesoporous carbons from coal tar pitch by KOH activation method using microwave and magnesium oxide (MgO) template [128]. Thus prepared carbon material shows high specific surface areas (1003–1394 m^2^ g^−1^) and also achieved high specific capacitance of 224 F g^−1^ in aqueous 6 M KOH electrolyte after 1000 cycles. Their results show that microwave-assisted KOH activation methods using the MgO template would represent a simple alternative to the production of cost-effective mesoporous carbon materials for energy storage applications. Similarly, they have also prepared micro-wave assisted high surface area mesoporous carbon materials by ZnCl_2_ activation [129]. High rate capability sustaining 184 F g^−1^ specific capacitance at 0.05 A g^−1^ after 1000 charging−discharging cycles was observed. Furthermore, the materials show an energy density of 4.94 Wh Kg^−1^ at a power density of 740 W kg^−1^ [129].

Pyrolysis of soft materials is another simple strategy to produce carbon materials. Kong and co-workers reported novel mesoporous carbons using this method [130]. They have pyrolyzed, polyacrylonitrile−*b*−polystyrene−*b*−polyacrylonitrile (PAN−b−PS−b−PAN) block−copolymers where PAN fraction generates a carbon network and release of PS after pyrolysis creates the mesopores resulting in the formation of surface areas and controlled mesopore size carbon materials. The prepared materials showed specific capacitance of 185 F g^−1^ at 0.625 A g^−1^ with remarkable cycle stability in aqueous electrolyte (2 M KOH) indicating the potential of direct pyrolysis of block-copolymer derived mesoporous carbon in energy storage applications. Similarly, Wang and co-workers reported a self-templated method and obtained mesoporous carbon material [131]. Here, they used carbon tetrachloride as a carbon precursor, which upon reduction by sodium–potassium alloy resulted in the formation of mesoporous carbon that exhibit high surface area and pore size distributions are rather narrow. Galvanostatic charge–discharge measurements revealed that the prepared carbon material achieves 259 F g^−1^ at 1 A g^−1^ and sustain 92% capacitance after 6000 charge−discharge cycles. 

**Figure 4:**
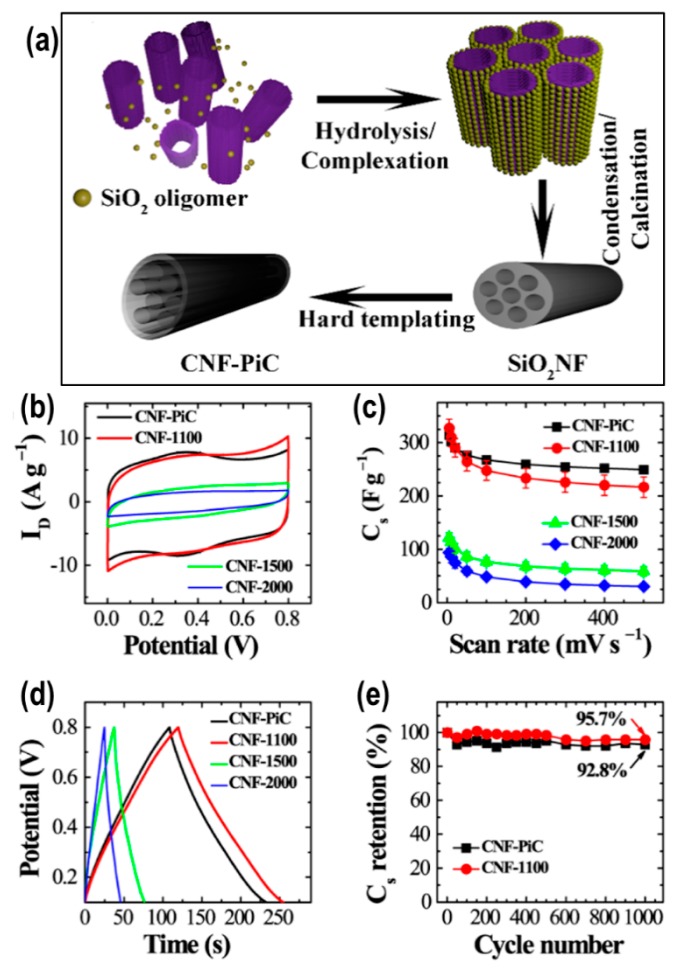
(**a**) Illustration of nanoporous carbons fibers prepared via carbonization and hard templating method and the electrochemical results. (**b**) CV performance of different samples at 50 mV s^−1^, (**c**) Capacitance values vs. scan rate for samples carbonized at different temperatures. (**d**) Galvanostatic charge−discharge performance at 1 A g^−1^. (**e**) Capacity retention performance recorded at 10 A g^−1^. Reproduced from Reference [132]. Copyright American Chemical Society, 2016.

Rodriguez-Abreu, Shrestha, and co-workers have synthesized carbon nanofibers (CNF) using nanocasting approach, where silica nanofibers obtained from chromonic liquid crystals (Figure 4) served as template [132]. Due to the randomly oriented graphitic layers and mesoporous structures in the carbon nanofibers, the material showed outstanding electrochemical supercapacitance performance. The high specific capacitance of 327 F g^−1^ was achieved at 5 mV s^−1^, which is far better than the capacitance of commercially available activated carbons. As we have mentioned before, the electrochemical performance of SCs depends on various parameters including the electrochemically accessible surface area of the electrode material, porosity distribution (common participation of micro- and meso-porosity), the interconnectivity of the pores, surface functional groups, conductivity and agglomeration of active material, and wettability level by the electrolyte. It should be noted that wettability by an electrolyte influences the electric cross resistance of the electrode and the level of electrolyte ion filling within the electrode inferring that the level of wettability is an important factor that influences the quantity of the accumulated charge in the electrode surface. Therefore, higher wettability enhances the area of the electrochemically accessible surface of the electrode material resulting in the high possibility of accumulating more charges and hence more energy can be stored. 

Rodriguez-Abreu, Shrestha, and co-workers have found that the carbon nanofiber shows high capacitance retention at high scan rates and high current densities [132]. Carbon nanofiber before heat-treatment (CNF−PiC) showed greater performance ca. 79% capacitance retention upon increasing scan rate from 5 to 500 mV s−1 (Figure 4c), which can be attributed to the better wettability of electrode due to the presence of surface oxygen-containing groups resulted from the synthesis process of the CNF−PiC. Polar surface functional groups on the carbon surface increase the wettability enabling the high capacitance retention in the CNF−PiC.

**Figure 5:**
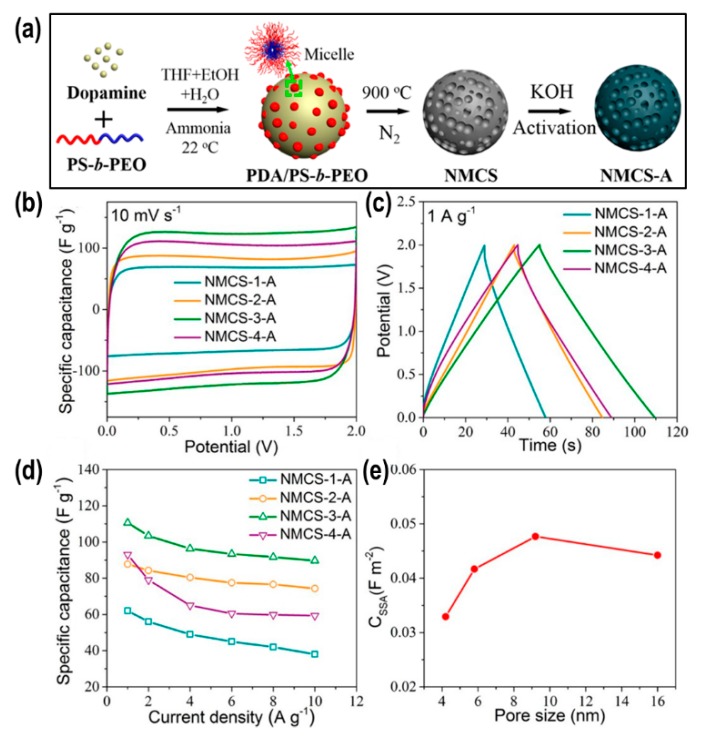
(**a**) Synthetic route to the fabrication of nitrogen-doped mesoporous carbon spheres (NMCS) followed by their activated products (NMCS−A) and electrochemical performances; (**b**) CV curves recorded at 10 mV s^−1^ as typical example, (**c**) CD curves at 1 A g ^−1^. (**d**) Calculated capacitance vs. current densities for different samples, and (**e**) Specific surface area normalized capacitance corresponding to 1 A g^−1^ current density vs. pore size. Reproduced from Reference [133]. Copyright American Chemical Society, 2017.

Nitrogen doping in the carbon matrix is another efficient way to design high energy storage supercapacitors due to the pseudocapacitive behavior of the nitrogen-doped carbons. Nitrogen-doped carbons also exhibit higher conductivity and contribute to enhancing the overall electrochemical performance of the materials. Recently, Yamauchi, Ariga, and co-workers have successfully prepared nitrogen-doped mesoporous carbon of sub-micrometer particle size (∼300 nm) in spherical shape using polymer micelles followed by KOH activation method and studied the electrochemical performance (Figure 5) [133]. They succeeded to precisely control the mesopore size from 4 to 16 nm by a subtle balance of di-block polymer, polystyrene−block−poly(ethylene oxide) (PS−*b*−PEO) templates and the material could achieve very high specific surface area (2000 m^2^ g^−1^). They have observed pore size is correlated with the degree of polymerization of the PS blocks of the polymer. They have fabricated symmetrical cells using these porous carbons and compared the electrochemical capacitive performance both in organic [1 M tetraethylammonium tetrafluoroborate/acetonitrile (TEA BF_4_/AN)] and ionic liquid [1−ethyl−3−methylimidazolium tetrafluoroborate (EMIMBF_4_)] electrolytes. The capacitive performance was found better in ionic electrolyte giving specific 170 F g^−1^ at 1 A g^−1^. In case of organic electrolyte specific capacitance was ca. capacitance of 111 F g^−1^. 

They have also performed a systematic study to underline the impacts of pore size distribution on the supercapacitive performance. They have determined the relationship between the electrochemical supercapacitance and the pore size of the series of nanoporous carbon spheres. To exclude the influence of specific surface area in the different carbon samples, they normalized the calculated specific capacitance by the specific surface area (*C*_SSA_) and plotted against the pore size (Figure 5e). As displayed in Figure 5e, the *C*_SSA_ increases with the mesopore size, attains a maximum, and decreases indicating greater utilization of electrode surface by the electrolyte ions in the sample that possess large mesopores. The decrease of the *C*_SSA_ after the maximum is caused due to the high fraction of micropores. Based on the experimental evidence they have concluded that that the carbon materials that have a low fraction of micropores with large mesopores are the best material to achieve the high specific surface area-normalized capacitance, *i.e.* to design the better supercapacitor systems compared to the conventional carbon materials. 

Recently, Wen et al. reported hierarchical porous 2D carbon sheets (surface area ca. 2788 m^2^ g^−1^) using pyrrole as carbon source and MgO as a template followed by KOH activation [134]. The prepared 2D carbon sheets consist of both micropores and mesopores with well-balanced pore size distribution and also display high conductivity. As a result, the material gave a high gravimetric specific capacitance of 226.4 F g^−1^ at 1 mV s^−1^ in a two-electrode cell configuration in an aqueous electrolyte (1 M H_2_SO_4_) followed by the exceptional cycle performance achieving 97% capacitance retention even after 10,000 cycles at 10 A g^−1^ demonstrating the promising potential of hierarchical 2D porous carbon sheets as electrode materials for advanced supercapacitors [134]. Similarly, Anandan and co-workers have recently reported the 3D ordered mesoporous carbons using KIT-6 and sucrose as template and carbon source, respectively [135]. The resulting carbon material possesses a specific area of 1017 m^2^ g^−1^ with a mean pore size of 4.1 nm and pore volume of 1.14 cc g^−1^ and achieved 252 F g^−1^ specific capacitance at 0.5 A g^−1^ followed by good cyclic performance retaining 91% capacitance for very long charge−discharge cycles of 30 000. The excellent electrochemical energy storage performance is the results of the 3D porous structure, high surface area, and interconnected porous structure which favor the rapid electrolyte ion transfer during the electrochemical process [135].

Lin, Zhang, and co-workers have reported a micelle-induced assembly method for the preparation of conductive porous carbon materials where they have used quantum dots (GQDs) as a carbon precursor and block copolymer F127 as template [136]. The prepared carbon material has interconnected mesoporous structure and thus exhibits high specific surface area of 1323 m^−2^ g^−1^ and high electrical conductivity of 73 S m^−1^, which enabled fast electron/ion transport especially when the mass loadings in supercapacitor electrode are high. In a three-electrode cell, the material shows 315 F g^−1^ specific capacitance at 1 A g^−1^ followed by good rate ability. About 54% capacitance was retained at 100 A g^−1^. Most importantly, they have found that even if the active mass of the material is high, 20 mg cm^−2^, at a current density of 10 A g^−1^, the material showed remarkably high areal capacitances ca. 2.8 F cm^−2^. The observed areal capacitance is better than reported values for other porous carbon materials. Furthermore, the device in the symmetric supercapacitor cell, showed maximum energy densities of 9.21 Wh kg^−1^ (2 mg cm^−2^) and 6.45 Wh kg^−1^ (20 mg cm^−2^) demonstrating the importance of the high surface area and interconnected porous structure of mesoporous carbons in the high energy density supercapacitors. 

### 2.4. Nanoporous Carbons from Natural Biomass 

In this section, we overview recent developments and advancement of the electrochemical energy storage performances of the porous carbons materials with hierarchical nanopores structure designed from various natural biomass and agricultural wastes by physical and chemical activation methods. Biomass-derived nanoporous carbon materials are attractive for their high surface area resulted due to hierarchical micro-, meso- and macro-porous architectures, good electrical conductivity, low-cost, and excellent electrochemical stability. Thus, nanoporous carbon materials derived from natural precursors with superior surface textural properties are highly desired candidates for the emerging nanotechnologies, particularly in energy storage related applications. 

All of the natural biomass or agricultural wastes contain lignocellulose, which produces char when pyrolyzed at moderate temperature in the air or nitrogen atmosphere. The char has a low specific surface area and hence such materials have limited practical applications due to the lack of porosity. Using direct carbonization or physical and chemical activation at appropriate carbonization temperatures and atmosphere porosity in the carbon network can be intentionally inserted. Large and industrial-scale nanoporous activated carbons can be prepared by physical activation method, where biomass or agro-wastes are carbonized in advanced are activated under constant flow steam or carbon dioxide at high temperatures (800−1100 °C). However, the low specific surface area of physically activated carbons was reported (500−1000 m^2^ g^−1^) and hence they have limited use in technological applications including high energy storage. 

Chemical activation method enhances the surface textural properties and activated carbons with very high surface area (>1000 m^2^ g^−1^) can be obtained rather easily. This method includes impregnation of starting precursor materials (bio-mass) with a dehydrating agent also called activating agent which could be salt, acid or alkali and then carbonized at relatively lower temperature ranges 400−800 °C under a constant flow of nitrogen or argon gas or inert atmosphere. Potassium chloride (KCl), zinc chloride (ZnCl_2_), phosphoric acid (H_3_PO_4_), sulfuric acid (H_2_SO_4_), sodium carbonate (Na_2_CO_3_), sodium hydroxide (NaOH), and potassium hydroxide (KOH) are the commonly employed activating agents, which favor pyrolytic decomposition of lignocellulose. Because of depolymerization and dehydration of biopolymers resulted in the enhancement of porosity. High specific surface areas are therefore expected in the chemically activated porous carbon materials. Carbonization temperature, hold time and impregnation ratio and starting precursor material itself are the key parameter that controls the porosity of the biomass-derived nanoporous activated carbon materials. In recent days, extensive studies have been focused to recycle technology and conversion of waste materials into functional carbons having high porosity, interconnected mesopores, and hierarchical micro- and meso-porous structures represent cost-effective ways to produce efficient materials in energy storage applications. Herein, we discuss some of the recent examples.

Shrestha, Rajbhandari, and co-workers have fabricated novel nanoporous carbon materials from agricultural waste locally called Lapsi, *Choerospondias axillaris* seed stone, by chemical activation with ZnCl_2_ at low carbonization temperature of 400 °C [137]. They have systematically investigated the effects of activation/carbonization conditions such as ZnCl_2_ impregnation ratio and carbonization time on the porosity of the derived carbons. They reported specific surface area and pore volume in the range of 697 to 1317 m^2^ g^−1^ and 0.45 to 1.2 cc g^−1^, respectively. The optimal sample showed good electrochemical performance. The high specific capacitance of 328 F g^−1^ at 5 mv s^−1^ was observed in 1 M H_2_SO_4_ showing the potential of low-temperature carbonization of Lapsi seed-derived nanoporous carbon materials in energy storage application. Further advancement in porosity and surface area was achieved by Shrestha and co-workers by H_3_PO_4_ activation method using corn cob as the carbon precursor [138]. Here again, they used low-temperature carbonation of 400 °C and best sample achieved specific surface area and pore volumes of 1288 m^2^ g^−1^ and 1.64 cm^3^ g^−1^, and the material performs well giving high specific capacitance of 340.8 F g^−1^ at 5 mV s^−1^ and also long cycle stability retaining 96% capacitance after 1000 charge−discharge cycles [138]. Using corn cob, Jiang, Lian, and co-workers have prepared 2D biochar nanosheets containing quinone and pyrone as pseudocapacitive oxygen functional groups through high-temperature thermal flash exfoliation method [139]. From the electrochemical measurements capacitance of the 2D biochar nanosheets was found to be 221 F g^−1^ although the material displays low surface area (543.7 m^2^ g^−1^). The high electrochemical performance of the material is the result of exfoliated porous carbon nanosheets. Rate performance of the materials was also good sustaining 78% of capacitance at a high current density of 40 A g^−1^ with almost no capacity loss after 5000 charging−discharging cycles. Their results demonstrated that the exfoliation method may be suitable for reducing the production cost of the carbon materials desired in energy storage applications. 

Shrestha and co-workers have also produced high surface area nanoporous carbon materials, using bamboo as a carbon source by the low-temperature H_3_PO_4_ action method [140]. Phosphoric acid activation of bamboo powder at 400 °C yielded porous carbon with a surface area and pore volume of 1431 m^2^ g^−1^ and 1.26 cm^3^ g^−1^, respectively. They have shown that the surface area and porosity can be tuned from 218 to 1431 m^2^ g^−1^ and 0.26 to 1.26 cm^3^ g^−1^, respectively, by simply changing the phosphoric acid mixing ratio with bamboo powder. Cyclic voltammetry and galvanostatic charge−discharge results revealed a high specific capacitance of 256 F g^−1^ at a scan rate of 5 mV s^−1^ with 92% capacitance retention after 1000 cycles. 

**Figure 6:**
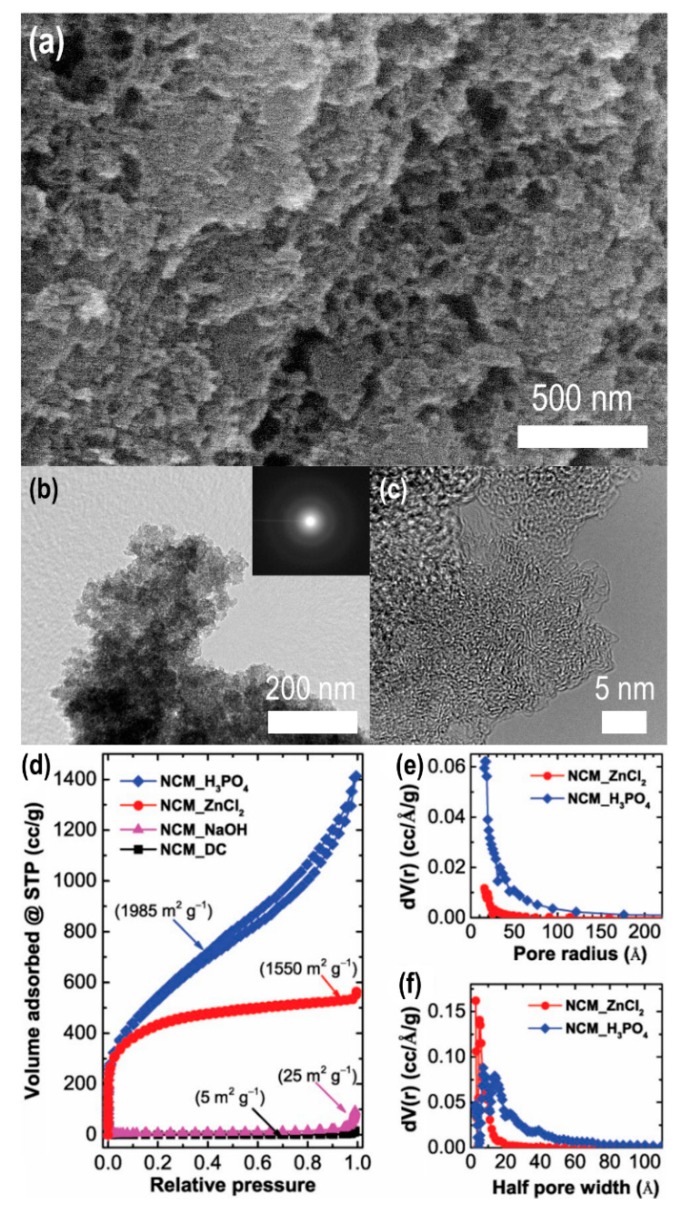
Electron microscopy observation and surface textural properties of activated nanoporous carbon materials (NCM) derived from chemical activation of Areca Catechu Nut. (**a**) SEM, (**b**) TEM and (**c**) HR−TEM images of H_3_PO_4_ activated NCM as typical example. (**d**) Nitrogen sorption isotherms of activated sample, for comparison isotherm for NCM_DC (directly carbonized) is also included. (**e**) Pore size distribution curves obtained from Barrett−Joyner−Halenda (BJH) method, and (**f**) Corresponding pore size distribution obtained from density functional theory (DFT) method. Reproduced from Reference [141]. Copyright Springer Science Business Media, 2017.

**Figure 7:**
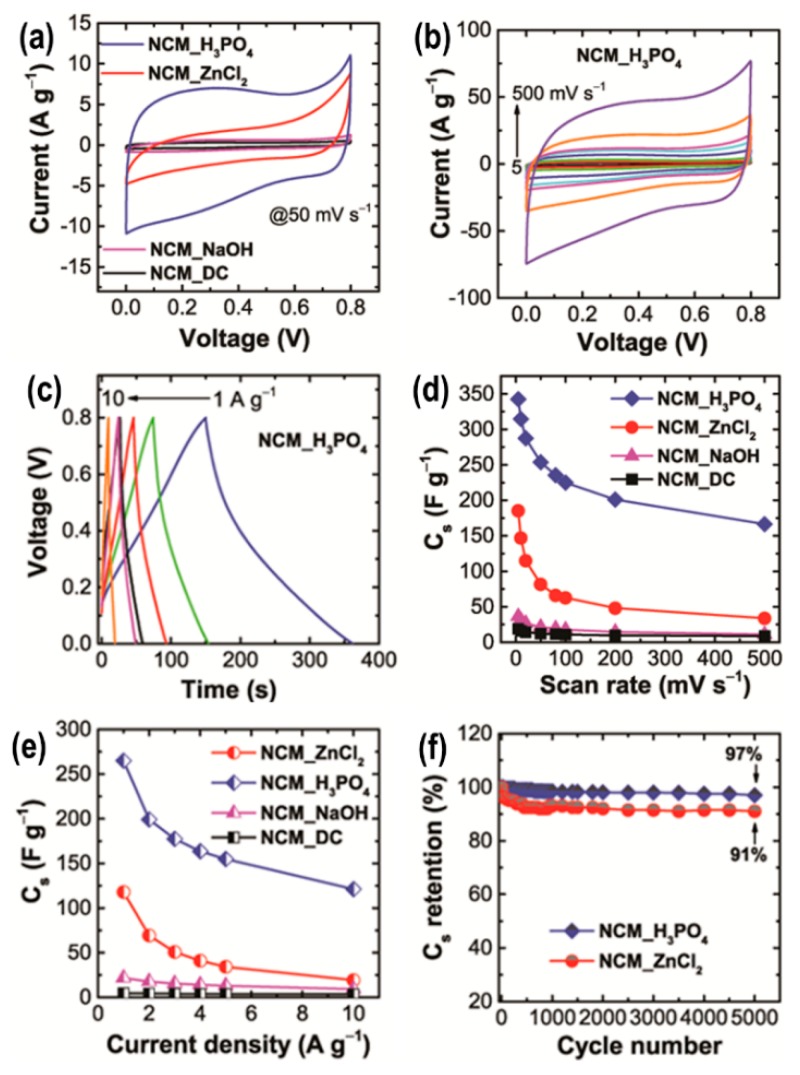
Electrochemical performance (cyclic voltammetry and chronopotentiometry) of activated nanoporous carbon materials (NCM) derived from chemical activation of Areca Catechu Nut (**a**) Comparison of CV curves for the prepared samples at 50 mV s^−1^, (**b**) CV curve vs. scan rate for NCM_H_3_PO_4_ characteristic of EDLC behavior, (**c**) Corresponding CD curve vs. current densities (1− 10 A g^−1^), (**d**) Calculated specific capacitances vs. scan rates for prepared samples, (**e**) Specific capacitances obtained from charge−discharge measurements vs. current densities, and (**f**) Charge−discharge cycle test at 10 A g^−1^ NCM_ZnCl_2_ and NCM_H_3_PO_4_. Reproduced from Reference [141]. Copyright © 2017, Springer Science Business Media.

They have also fabricated high surface area nanoporous carbon material (NCM) from novel precursor Areca Catechu Nut, which was chemically activated at 400 °C by different activating agents such as NaOH, ZnCl_2_, and H_3_PO_4_ (Figure 6) [141]. They have found that the obtained carbon materials have an amorphous structure. Depending on the activating agent used the NCM contains macropores (NaOH activation), micropores (ZnCl_2_ activation), and hierarchical micro- and meso-pore (H_3_PO_4_ activation) architectures (Figure 6d). Surface area and pore volume were found in the range of 25 to 1985 m^2^ g^−1^ and 0.12 to 3.42 cm^3^ g^−1^, respectively The best sample obtained by H_3_PO_4_ activation showed very good electrochemical supercapacitive performance in 1 M H_2_SO_4_ giving specific capacitance of 342 F g^−1^ at a scan rate of 5 mV s^−1^ followed by long cycle stability retaining 97% capacitance after 5000 charge−discharge cycles (Figure 7).

Zheng, Liu, and co-workers have demonstrated the first example of 3D honeycomb-like hierarchically-structured carbon [142]. They have used sewage sludge as a carbon precursor that contains high-ash. They have concluded that because of the fly-silicon process honeycomb-like hierarchical structures are formed. This newly synthesized carbon material displayed novel honeycomb-like frameworks and interconnected pores. As a result, the material achieved a high specific surface area of 2839 m^2^ g^−1^ and a large pore volume of 2.65 cm^3^ g^−1^. Electrochemical measurements showed that the honeycomb-like hierarchically-structured carbon material exhibit a high specific capacitance of 379 F g^−1^ at a current density of 0.5 A g^−1^. Long term cycle stability test revealed 90% capacitance retention after 20,000 cycles at 20 A g^−1^. Furthermore, they have observed an energy density of 30.5 W h kg^−1^ in the symmetric supercapacitor system in an aqueous solution, which is high among previously reported values. Their results show that sewage sludge-derived carbons can be suitable electrode materials for high energy density supercapacitors.

Wei, Gao, and co-workers have reported Batata leaves and stalks derived three-dimensionally interconnected carbon nanorings consisting of multilayer of graphenes from 3 to 16 layers [143]. They have also observed that this new type of carbon also contains hetero atom O and N containing surface functional groups, which increases the wettability and conductivity of the materials. The prepared carbon nanorings exhibit a very high BET surface area of 3114.74 m^2^ g^−1^. Graphitic carbon structure together with three-dimensionally interconnected nanoring networks makes the material with appropriate pore size matching well with electrolyte ions. The electrochemical energy storage capacity of their material was excellent. The specific capacitance of 532.5 F g^−1^ was achieved at 1 A g^−1^. Only 5% capacitance was lost after 1000 cycles at 1 A g^−1^ and the electrode exhibits an energy density of 25.8 Wh kg^−1^ in 1 M H_2_SO_4_ aqueous acid electrolyte. Interestingly, the materials also showed excellent capacitive performance in an aqueous alkaline electrolyte (6 M KOH). Specific capacitances of 350.0 F g^−1^ was achieved at 1 A g^−1^, which was accompanied by excellent cycling stability retaining 95% capacitance retention after 1000 cycles. The electrode also possesses a comparable energy density of 24.5 Wh kg^−1^ in alkaline electrolytes [143]. 

Using corncob sponge, Fan and co-workers recently reported nitrogen and sulfur co-doped three-dimensionally interconnected honeycomb-like porous carbons through a simple one-pot carbonization and activation method [144]. The prepared binary heteroatom doped carbon material showed a high surface area of 1874 m^2^ g^−1^ due to 3D honeycomb-like highly porous framework structure. Due to the Faradic contribution of hetero atoms doping together with EDLC contribution of carbon, the prepared materials achieved high specific capacitance of 404 F g^−1^ at a current density of 0.1 A g^−1^ in an aqueous 6 M KOH electrolyte. The supercapacitor electrode loses only 1% capacitance after 10,000 charging−discharging cycles. They have also recorded an energy density of 30 W h kg^−1^ at 8 kW kg^−1^ from the symmetric flexible solid-state supercapacitor cell in a PVA/KOH gel electrolyte [144]. Similarly, Zhang and Yang have reported the synthesis of corncob-derived carbon with hierarchical porosity through carbonization followed by KOH activation process, special biogenetic textures of corncob could be inherited, with pore size in the range of macro to microscale [145]. The specific surface area was ca. 1471.4 m^2^ g^−1^ and the material achieved specific capacitance of 293 Fg^−1^ at 1 Ag^−1^. They have also assembled supercapacitor devices and recorded quite good value of energy density ca. 20.15 Wh kg^−1^ at power densities of 500 Wh kg^−1^ and only 0.1% capacity was lost after 4000 charging−discharging cycles [145]. Very recently, Zheng and co-workers have reported nitrogen-doped 2D carbon nanosheets using peanut dregs, soybean meal, and rapeseed dregs as carbon sources by KOH-free method [146]. They have introduced the saponification method to regulate the microstructure and components of carbon materials for the first time and novel mild activation agents such as potassium oxalate monohydrate and dicyandiamide were used to activate the char, which resulted in the formation of carbon nanosheet with high surface area (2470 m^2^ g^−1^). Following this simple strategy, they have made high nitrogen content (4.96%) 2D carbon nanosheets. Owing to the high specific surface area, 2D bilayer structure, hierarchical porous architecture, and high nitrogen content, the prepared materials exhibit remarkable electrochemical performances. Specific capacitance was ca. 340 F g^−1^ at a current density of 0.5 A g^−1^ with excellent rate capability retaining a high capacitance of 282 F g^−1^ at a very high current density of 50 A g^−1^ without any loss of capacitance attenuation after 20,000 charge−discharge cycles in 6 M KOH aqueous electrolyte. In the symmetrical cell, they successfully recorded a maximum energy density of 55.5 W h kg^−1^ at 369 W kg^−1^ in 1.0 M LiPF_6_ electrolyte [146]. Hierarchical micro-mesoporous carbon (HPC−2) derived from Sichuan pepper has recently been reported by Zhang and coworkers [147]. In the first step, they pre-carbonized the precursor and then KOH activated at high temperature, which resulted in high porosity carbon with the surface area of 1823.1 m^2^ g^−1^. The Sichuan pepper-derived carbon material display reasonably good electrochemical performance with specific capacitance 171 F g^−1^ at 1 A g^−1^ without any capacitance loss until 10,000 charging−discharging cycles. KOH activation of coffee powder (waste material) interestingly yielded nitrogen-doped carbon as reported by Gupta and coworkers [148]. For the successful nitrogen doping coffee powder required to treat with melamine prior to the activation. The surface area of the resulting nitrogen-doped carbon was found to be 1824 m^2^ g^−1^ and the specific capacitance of 148 F g^−1^ was found at 0.5 A g^−1^. They have also fabricated a symmetrical coin cell device and recorded the highest specific capacitance of 74 F g^−1^ at a current density of 1 A g^−1^. Furthermore, the device exhibits an energy density of 12.8 Wh kg^−1^ at 6643 W kg^−1^ followed by 97% capacitance retention and maintaining 100% coulombic efficiency after 10 000 charging−discharging cycles. 

Xiao and coworkers have used high-temperature alkaline activation method and obtained nitrogen-doped (3.2%) carbon with hierarchical meso/microporous architecture from bamboo fungus [149]. The porous carbon featured honeycomb-like cellular framework with the rational distribution of mesopores and micropores and exhibits a high specific surface area (1708 m^2^ g^−1^). In the symmetric supercapacitor cell, the prepared material exhibits an energy density of 4.3 Wh kg^−1^ with negligible degradation of the capacitance for 10 000 charge−discharge cycles at 10 A g^−1^ [149]. 

Zhong and co-workers have also demonstrated very recently high nitrogen content (>8 atom %) nitrogen-doped mesoporous carbons from biomass [150]. In their study, they have used Peach gum and urea and polyethyleneimine as carbon and nitrogen sources, respectively. The prepared mesoporous material comprises a graphitic carbon framework structure. The surface area and conductivity of the carbon material were ca. 1161.4 m^2^ g^−1^ and 11.43 S cm^−1^, respectively. Owing to the high nitrogen content, high conductivity, high surface area, and hierarchical porous structure high fraction of mesopore, the prepared material showed excellent supercapacitance performance. The specific capacitance of 426 F g^−1^ was achieved at a current density of 0.5 A g^−1^ followed by ca. 3% capacitance loss in 10,000 charging−discharging cycles at 20 A g^−1^. Furthermore, in an assembled symmetric supercapacitor cell they have achieved excellent energy density ca. 30.28 Wh Kg^−1^ at 180 W Kg^−1^ in an aqueous electrolyte (1 M Na_2_SO_4_) [150]. 

These aforementioned examples of the biomass-derived hierarchical nanoporous carbons show the importance of the specific surface area, appropriate pores size distribution, nitrogen content, and electrical conductivity of the electrode material for the high-performance supercapacitors applications. The electrochemical performance of some of the biomass-derived nanoporous carbon materials is summarized in Table 1. Because of the easy availability and simple and cost-effective fabrication method, industrial-scale production of nanoporous carbon from biomass is possible, which will be advantageous for the large-scale commercial application of high energy storage supercapacitors.

## 3. Conclusions and Future Outlook

In this short review article, various hierarchical nanoporous carbons with high and well-developed porosity and high surface areas that are required for the high-performance supercapacitor devices have been overviewed. We have highlighted the promising scope of accessing nanoporous graphitic carbon materials from various carbon sources including: (i) direct conversion of self-assembled crystalline fullerene nanomaterials and metal−organic frameworks, (ii) hard- and soft-templating routes, and (iii) the direct carbonization and/or activation of biomass or agricultural wastes as non-templating routes. We have discussed several examples of different synthetic carbon sources or natural precursor raw-materials derived nanoporous carbon materials in energy storage applications. Various natural biomass or agricultural wastes containing cellulose, hemicellulose, and lignin are the potential carbon sources, which upon chemical activation at moderate temperature range (400−800 °C) generate micro- and meso-porous architecture in the carbon backbone. The mixing ratio of activating agent, carbonization temperature, and nature of the biomass itself are the tunable parameters for enhancing the surface area and porosity of the derived nanoporous carbon materials. 

Fullerene crystals-derived nanoporous carbon materials achieved a high specific capacitance up to 360 F g^−1^ at a scan rate of 5 mV s^−1^ followed by excellent cycling stability without any capacity loss after 10,000 cycles [109]. Metal−organic frameworks derived porous carbon material exhibited the very high specific surface area (2872 m^2^ g^−1^) and large pore volume (2.06 cc g^−1^) as a result the electrode achieved a high specific capacitance of 312 F g^−1^ at 1 mV s^−1^ [112]. Nanoporous carbon fibers produced by templating silica fibers obtained from chromonic liquid crystals as a template could deliver a high specific capacitance of 327 F g^−1^ at a scan rate of 5 mV s^−1^ [132]. Similarly, due to the interconnected mesopore structure and high electrical conductivity, porous carbon materials obtained using graphene quantum dots as carbon source and block copolymer as a template achieved a high specific capacitance of 315 F g^−1^ at 1 A g^−1^ [136]. Depending on the carbon source and preparation method, biomass-derived nanoporous carbon materials also showed excellent electrochemical supercapacitance. Due to the three-dimensionally interconnected carbon nanorings structure, the Batata leaves and stalks-derived carbon materials showed a very high specific capacitance of 532.5 F g^−1^ at 1 A g^−1^ [143].

These recent examples of the electrochemical supercapacitance performance of the porous carbon materials demonstrate the importance of various key parameters that contributes to enhancing the electrochemical supercapacitance performance. Some of the important parameters include high porosity, pore size distribution, high electrochemically accessible surface area, hierarchical micro/mesopore architecture with interconnected mesopores, nitrogen-doping in the carbon matrix, graphitic framework structure, the conductivity of the electrode materials, and wettability. Such carbons represent the excellent materials required for excellent performance (high specific capacitance, high rate capability, long cycle life, and high energy density) of supercapacitors. However, it is yet challenging to fabricate carbon materials that possess all these characteristic feathers. One strategy would be the fabrication of novel composite materials for hybrid supercapacitors comprising of high electrochemical surface area nitrogen-doped carbon materials with hierarchical micro/mesopore architecture with interconnected mesopores and excellent pseudocapacitive metal oxides. The synergy from both the electrical double layer and pseudocapacitive materials would lead to an unexpected performance in energy storage.

The novel nanomaterial fabrication concept, nanoarchitectonics, could be extended to design such high performance nanoporous graphitic carbon materials with tunable surface area, pore volume, nanopores engineering, or control of hierarchical nanoporous structure, which could be an asset in the future to develop new technology for high energy storage supercapacitors. 

## Figures and Tables

**Table 1:** Electrochemical supercapacitance performance of biomass-derived various nanoporous carbon materials.

Biomass	Electrolyte	Current Density/Scan Rate	Specific Capacitance (F g^−1^)	Reference
Batata leaves and stalks	1 M H_2_SO_4_	1 A g^−1^	532.5	[143]
Batata leaves and stalks	6 M KOH	1 A g^−1^	350	[143]
Peach gum	6 M KOH	0.5 A g^−1^	426	[150]
Peanut dregs	6 M KOH	0.5 A g^−1^	340	[146]
Corncob	6 M KOH	0.1 A g^−1^	404	[144]
Areca catechu nut	1 M H_2_SO_4_	5 mV s^−1^	342	[141]
Bamboo	1 M H_2_SO_4_	5 mV s^−1^	256	[140]
Corncob	1 M H_2_SO_4_	5 mV s^−1^	340.8	[138]
Sewage sludge	1 M Na_2_SO_4_	0.5 A g^−1^	379	[142]
Corn cob	0.5 M H_2_SO_4_	0.5 A g^−1^	210	[139]
Lapsi seed	1 M H_2_SO_4_	5 mV s^−1^	328	[137]
